# Professional long distance runners achieve high efficiency at the cost of weak orbital stability

**DOI:** 10.1016/j.heliyon.2024.e34707

**Published:** 2024-07-16

**Authors:** Siddhartha Bikram Panday, Prabhat Pathak, Jooeun Ahn

**Affiliations:** a Division of Sports Industry and Science, Hanyang University, Republic of Korea; b Department of Art and Sportainment, Hanyang University, Republic of Korea; c John A. Paulson School of Engineering and Applied Sciences, Harvard University, USA; d Department of Physical Education, Seoul National University, Republic of Korea; e Institute of Sport Science, Seoul National University, Republic of Korea

**Keywords:** Long distance running, Efficiency, Orbital stability, Cost of transport, Floquet multipliers

## Abstract

Successful performance in long distance race requires both high efficiency and stability. Previous research has demonstrated the high running efficiency of trained runners, but no prior study quantitatively addressed their orbital stability. In this study, we evaluated the efficiency and orbital stability of 8 professional long-distance runners and compared them with those of 8 novices. We calculated the cost of transport and normalized mechanical energy to assess physiological and mechanical running efficiency, respectively. We quantified orbital stability using Floquet Multipliers, which assess how fast a system converges to a limit cycle under perturbations. Our results show that professional runners run with significantly higher physiological and mechanical efficiency but with weaker orbital stability compared to novices. This finding is consistent with the inevitable trade-off between efficiency and stability; increase in orbital stability necessitates increase in energy dissipation. We suggest that professional runners have developed the ability to exploit inertia beneficially, enabling them to achieve higher efficiency partly at the cost of sacrificing orbital stability.

## 1 Introduction

Marathoners need to run fast and efficiently to win the race. To achieve the desired speed with sufficient efficiency, they actively adjust their stride frequency and stride length, adopting proper pacing strategies [[1], [2], [3]]. The adequate pace enables the runners to maintain homeostasis and minimize segmental variability throughout the run [4,5]. Multiple studies have directly shown that professional marathoners who can exploit their optimal pace run more efficiently than non-athletes or recreational runners [[6], [7], [8], [9]]. Also, previous studies have shown that elite marathoners can maintain a constant cost of transport (COT) over a wider range of speeds than novices, indicating that elite athletes can maintain higher running efficiency over a broader range of running speed [[10], [11], [12], [13], [14]].

Previous studies also suggest that professional runners can consciously or subconsciously adjust their running strategy in response to physiological demands, and the ability of adjustment at least partly depends on experience and training. Hunter and Smith found that elite marathoners, even when they are fatigued, alter their running kinematics to keep the metabolic cost close to the optimal value which a curve fitting model estimates [9]. It was additionally reported that elite runners, despite kinematic differences, regulate their foot contact time and apparent leg impedance to maintain the resultant metabolic expenditure at approximately optimal level [15,16]. Compared with well-trained runners, novice runners show a greater disparity between their stride frequency and the optimal value that a curve fitting model computes [8].

Energy efficiency is a critical factor in endurance running, but it is not clear whether the importance of efficiency precedes that of stability. Weak stability of running can result in a large deviation from planned kinematics under unexpected perturbation, potentially causing falls or jeopardizing homeostasis and degrading performance. Therefore, professional runners as well as recreational joggers must pursue both stability and efficiency. However, stability and efficiency can hardly be increased simultaneously; rather, there exists a clear trade-off between the two [17]. Stability or asymptotic convergence to the planned kinematics under perturbation inevitably requires energy dissipation. The faster convergence or stronger stability accordingly implies greater dissipation of the energy or inefficiency. For example, in the case of pure rolling of a disk, which is the extremely efficient form of locomotion, the stability of the resulting motion becomes marginal. In contrast, if the locomotion is controlled in such a way that its stability is extremely strong, preventing any perturbation from affecting the following strides, the entire system must consume or dissipate significant amount of energy [18].

Despite this coexistence of the demand for both efficiency and stability in running and their trade-off, few studies addressed the relative importance of the two. Past studies have focused only on a single aspect of motor performance (either efficiency or stability) to quantify the training-induced changes in the long-distance running [19,20]. Although a previous study confirmed the trade-off between efficiency and stability for assisted walking [21], no study addressed this important trade-off in long distance running, which obviously requires both efficiency and stability. Running consumes larger energy under plausibly larger internal and external perturbations than walking, and it is highly plausible that there exists the inevitable trade-off between the efficiency and stability in running regardless of the individual’s level of training. Therefore, it is probable that well-trained runners need to sacrifice stability to maintain high efficiency. On the other hand, considering the effect of motor learning of athletes, professional runners might achieve both high efficiency and strong stability despite the trade-off between the two. To investigate which of the two is valid, we formulated and tested this hypothesis: professional long-distance runners have higher efficiency and lower stability during running than novice runners.

Energy efficiency can be quantified in two primary ways: physiological and mechanical efficiency [22]. Physiological efficiency is typically measured by calculating the cost of transport (COT), which represents the O_2_ quantity required to move the body per distance [23,24]. COT provides a speed-independent, comprehensive measure of energy expenditure per distance, facilitating accurate performance predictions and inter-individual comparisons [12]. To quantify mechanical efficiency, we measured normalized mechanical energy (*E*_*norm*_), the ratio of total mechanical energy of the whole body to the kinetic energy assigned to the translation of the center of mass (COM). This ratio indicates how efficiently runners transform the total mechanical energy obtained from the metabolism into the energy of moving forward [25]. Excessive range of motion of each body segment during running, which results in more mechanical energy, requires more metabolism. Therefore, minimizing unnecessary segment motion is essential for maintaining high energy efficiency and achieving optimal running performance [9,22,25]. Lower *E*_*norm*_ indicates more efficient employment of degrees of freedom (DOFs), resulting in the decreased unnecessary energy usage consumed by superfluous limb movements [26].

To test the formulated hypothesis, we also evaluated the orbital stability, which refers to a system’s capability of returning to a limit cycle under a perturbation [[27], [28], [29], [30]]. The index of orbital stability is the Floquet multiplier (FM) that assesses how fast the perturbed trajectory asymptotically returns to the original limit cycle [31,32]. Numerous studies demonstrated that FM can robustly quantify changes in stability of human locomotion [[27], [28], [29], [30],^33^]. FM exhibits greater specificity in response to different perturbation conditions compared to other measures, making it useful for identifying particular types of instability induced by specific perturbations [33]. Considering the rhythmic nature of running, we quantified stability during long-distance running by performing the return map analysis and estimating the maximum magnitude of FM (max FM). A system is stable if the effect of any perturbation diminishes as time increases, and the strength of the stability can be quantified by the rate of the convergence. For a periodic system to have orbital stability, the max FM should be less than unity. A small magnitude of max FM indicates fast convergence or strong orbital stability. To investigate how well-trained runners balance between the two conflicting factors of stability and efficiency, we compared max FM, COT and *E*_*norm*_ of professional runners with those of non-athletes.

## 2 Methods

### 2.1 Participants

Consulting a study which compared the efficiency of professional runners and novices [8], we set the effect size as 1.97. We subsequently set statistical power and significance as 0.95 and 0.05, respectively. Using the chosen values of the effect size, power and significance, G*Power software (Heinrich Heine University, Dusseldorf, Germany) computed required sample size as 8 for each group. Accordingly, we recruited 8 professional runners (age: 28.6 ± 3.7 years, half marathon record: 76 ± 5.7 min, experience: 8.6 ± 4.4 years, weight: 66.3 ± 10.8 kg, height: 176.9 ± 8.2 cm) and 8 novices (age: 23.2 ± 1.7 years, weight: 76.7 ± 4.8 kg, height: 180.5 ± 7.6 cm). The criteria for participating as a professional runner are 1) a minimum of 5 years of professional training, 2) a coverage of more than 40 km each week, and 3) the ability to run on a treadmill [34]. Those who consistently exercise in aerobic activity for at least 2 h per week and did not have any disease could participate in the experiment as novices. We conducted the experiment according to the ethics established in the Declaration of Helsinki. The protocol of the study was approved by the institutional review board of Seoul National University on October 23 in 2020 (institutional review board number 2010/003–013). We informed participants about the entire procedure and collected written informed consent before the experiment.

### 2.2 Experimental protocol and setup

Participants were instructed to avoid any activity which could possibly induce fatigue for a minimum of 24 h prior to participation. A brief interview was conducted to gather demographic data and running history from each participant. Anthropometric measurements, including weight, height, age and shoe size were recorded. Following the interview and data collection, each participant completed a 10-min warm-up session on a treadmill (Any fitness. Ltd, Korea), which featured a large surface area (length: 250 cm, width: 120 cm), high maximum power (5 hp), and a high maximum belt speed (30 km/h). These specifications were chosen to minimize spatial constraints and reduce gait-phase dependent variation of the speed of the treadmill belt considering that reduced variation can diminish disparity between treadmill and overground running [35,36].

*Estimation of Preferred Running Speed (PRS):* PRS for each participant was estimated using the Up-Down method, described in previous studies [37,38]. We opted PRS over fixed speeds in our research design as it offers a personalized and physiologically relevant approach to assessing running intensity, ensuring comfort and efficiency for each participant [39]. Additionally, self-selection has been shown to promote endurance at higher intensity levels [40], aligning with the principle of biological systems striving to optimize performance while minimizing energy expenditure [41]. The PRS estimation process began with letting participants run at the warm-up speed. We gradually escalated the belt speed by 0.1 km/h till the participant identified a comfortable pace. Subsequently, we raised the belt speed by 2 km/h. After that, we gradually decreased it by 0.1 km/h till the participant determined their PRS. We covered the treadmill’s monitor to prevent participants from viewing their speed, minimizing potential reporting bias [38]. We repeated this procedure thrice, and calculated the mean value as PRS. We let participants report their PRS only when they recognized their stride length and rate as comfortable. Communication regarding PRS was facilitated through sign language (thumbs up/down for increase/decrease or an OK sign for the preferred speed). To secure safety by monitoring the exerted intensity, we calculated the reference maximal heart rate (HR) of each participants using the Fox’s equation, which is shown to be robust for measuring the maximal HR for diverse population [42,43].

**Fig. 1:**
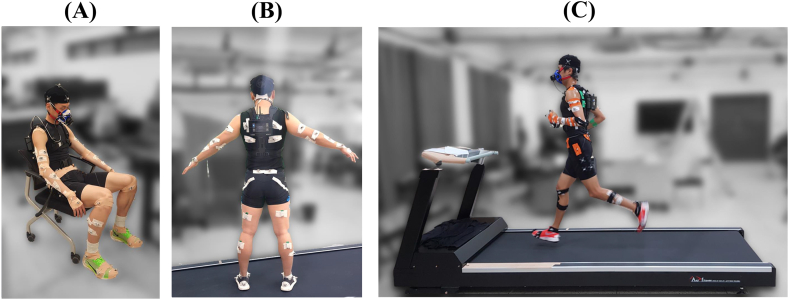
**Experimental Setup.** (A) Participant in a seated position with sensors affixed, getting prepared to measure steady-state O_2_ consumption. (B) Rear view of the participant standing for static trial, displaying the indirect calorimetry system as well as affixed retroreflective markers for motion capture. (C) Participant running on a treadmill with full setup, demonstrating data collection during main experiment.

After determining participants' PRS, we attached an indirect calorimetry device (K5, Cosmed, Italy) for estimating the amount of participants' metabolic energy expenditure as shown in Fig. 1(A–C). We operated the device according to a predetermined protocol [44] to monitor O_2_ inhalation and CO_2_ exhalation on a breath-by-breath basis. This instrument is known to yield standard errors below 1.6 percent and 2.2 percent for the measurement of O_2_ inhalation and CO_2_ exhalation rates respectively. Prior to operation, we calibrated the device using a cylinder with known O_2_ and CO_2_ content. Participants were fitted with the HR monitor, secured to the chest with a belt in a seated position. Before the main experiment, we conducted an 8-min protocol (5 min sitting, 3 min standing) to measure steady-state O_2_ consumption by instructing participants to sit and stand comfortably while monitoring the respiratory exchange ratio (RER) and HR, ensuring stability for more than 2 min, and extending the duration if fluctuations were observed [45].

**Fig. 2:**
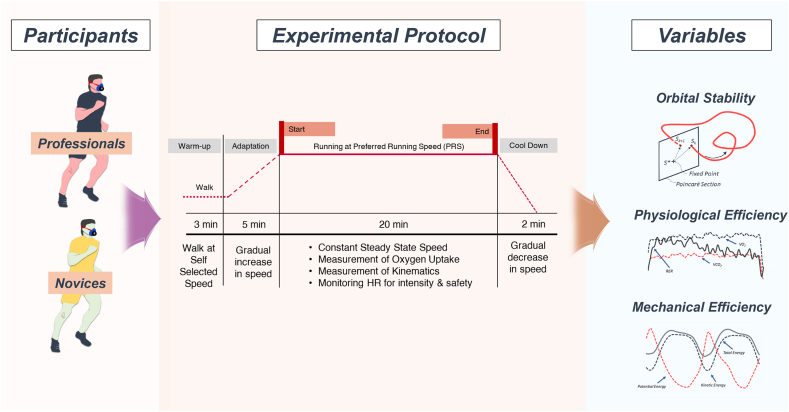
**Schematic of the research design.** Protocols of experiment, and computed variables for quantifying efficiency and stability are illustrated.

*Main Experiment:* The procedure of the main experiment is summarized in Fig. 2. At the beginning of the running experiment, participants walked for at least 3 min at their own pace. The speed was then steadily increased till it reached the PRS within 5 min. For a total of 20 min, participants ran at their PRS while O_2_ uptake and kinematics were recorded. To ensure safety, we continuously monitored HR in real-time [42,43]. A trial was immediately discontinued whenever the HR values exceeded the predicted maximum or upon request. After running for 20 min, participants were asked to cool down by walking at the self-selected speed for 2 min.

For kinematic analysis, we attached spherical retroreflective markers (12.7 mm) at anatomical bony landmarks:

⁃ Head: right/left temple, forehead and vertex
⁃ Arms: right/left medial/lateral head of the metacarpal, right/left radius/ulna-styloid process, right/left medial/lateral epicondyle of the humerus and right/left scapula-acromioclavicular joint
⁃ Lower extremities: right/left head of the 1st metatarsus, right/left proximal medial phalanx, right/left head of the 5th metatarsus, right/left posterior surface of the calcaneus, right/left fibula apex of the medial/lateral malleolus, right/left femur medial/lateral epicondyle, right/left great trochanter and right/left anterior/posterior superior iliac spine

Additionally, 4 markers were attached to the trunk, and 3 markers were attached to each of the shanks, thighs, forearms and upper arms for tracking. Thus, 58 spherical retroreflective markers were attached to each participant.

We tracked the retroreflective markers using 12 cameras (Optitrack Prime13, NaturalPoint, Inc., Oregon, USA) at 100Hz. We calibrated the system until the error went below 0.5 mm. We adopted the coordinate recommended by the International Society of Biomechanics, in which X, Y, and Z axes respectively correspond to the medial-lateral, anterior-posterior, and vertical coordinates of retro-reflective markers. A static trial with arms raised and feet positioned shoulder-width apart was captured to accurately reconstruct body segments for the Visual 3D model.

### 2.3 Data analysis

We applied a zero-lag 2nd order low-pass Butterworth filter on raw coordinates of the reflective markers; the cut-off frequency was set to 10 Hz [46,47]. Then, we generated a kinematic model of the entire body using Visual 3-D software. The kinematic model consisted of the shanks, thighs, feet, hands and forearms, upper arms, pelvis, trunk, and head. A mid-point estimation approach was used to locate the center of each joint [48].

Considering that we obtained the indirect calorimetry data through the breath-by-breath method, we filtered the raw data using a zero-lag 4th order low-pass Butterworth filter with the cut-off frequency of 0.04 Hz [49]. Then, we estimated O_2_ consumption (${\dot{V}O}_{2}$) and CO_2_ generation (${\dot{V}CO}_{2}$) based on the filtered data.

#### 2.3.1 Spatiotemporal variables during running

We estimated the time point of the heel strike using a method by Smith et al., who identified the initiation of heel strike as the moment when the vertical distance between the heel and posterior superior iliac spine reaches the maximum [50]. Then, we evaluated stride length as the distance that the treadmill belt traveled between the proximal end positions of a foot at heel strike and the next ipsilateral heel strike. Likewise, we computed stride time as the interval between successive ipsilateral heel strikes. To estimate three dimensional angles of the hips, knees, and ankles, we used the Cardan sequence [51].

#### 2.3.2 Mechanical efficiency

We first estimated the total mechanical energy during running as the sum of the potential energy and the kinetic energy of all segments. We estimated the inertial properties of each segment consulting previous studies [[52], [53], [54]]. Then, we calculated the participant’s total mechanical energy using the following equation:

(Eq. 1) $$E_{mech}=\sum_{i=1}^{15}m_{i}{gh}_{i}+\sum_{i=1}^{15}\frac{m_{i}v_{i}^{2}}{2}+\sum_{i=1}^{15}\frac{\emptyset_{ix}\omega_{ix}^{2}}{2}+\frac{\emptyset_{iy}\omega_{iy}^{2}}{2}+\frac{\emptyset_{iz}\omega_{iz}^{2}}{2}\text{,}$$

where $h_{i}$, $v_{i}$ and $m_{i}$ are the height of COM, the velocity of COM, and the mass of $i^{\text{th}}$ segment, respectively. The symbols $\emptyset_{ix}$, $\emptyset_{iy}$ and $\emptyset_{iz}$ are the moments of inertia of $i^{\text{th}}$ segment about frontal, sagittal and longitudinal axis, respectively, and the symbols $\omega_{ix}$, $\omega_{iy}$ and $\omega_{iz}$ are the angular velocities of $i^{\text{th}}$ segment about frontal, sagittal and longitudinal axis, respectively. We approximated the gravitational acceleration, g as 9.81 m/s^2^.

We also calculated the translational kinetic energy of the COM as

(Eq. 2) $$E_{trans}=\frac{Mv_{COM}^{2}}{2}\text{,}$$

where M and v are the total body mass and the velocity of COM. Then, the normalized mechanical energy, *E*_*norm*_ was calculated as:

(Eq. 3) $$E_{norm}=\frac{E_{mech}}{E_{trans}}\text{.}$$

A smaller value of *E*_*norm*_ indicates higher ability to obtain the translational kinetic energy from the whole-body motion or higher mechanical efficiency during running.

#### 2.3.3 Physiological efficiency

We monitored RER, which is defined in Eq. (4) to be within 0.70–0.90 to ensure that the participants ran under the submaximal exercise intensity [55].

(Eq. 4) $${\text{RER}}_{time\;intervals}=\frac{1}{n}\sum_{t=1}^{n}{\left(\frac{{\dot{V}CO}_{2}run}{{\dot{V}O}_{2}\;run}\right)}_{t}\text{,}$$

where ${\dot{V}O}_{2}run$ and ${\dot{V}CO}_{2}run$ are the volume of O_2_ consumed and CO_2_ produced in milliliter per time (*t*), respectively for each of the time intervals during running trial.

To evaluate COT as the measure of physiological efficiency, we evaluated the average steady-state values of ${\dot{V}O}_{2}$ during sitting as the baseline;

(Eq. 5) $$mean{\dot{\;V}O}_{2}rest=\frac{1}{n}\sum_{t=1}^{n}{\left({\dot{V}O}_{2}\;rest\right)}_{t}\text{.}$$

Then, COT during running was calculated as

(Eq. 6) $${mean\;\mathrm{COT}}_{time\;intervals}=\frac{1}{n}\sum_{t=1}^{n}{\left(\frac{{\dot{\;V}O}_{2}run-mean\;{\dot{V}O}_{2}rest}{mv}\right)}_{t}\text{,}$$

where m and v represent the body mass of the participant and the participant’s PRS. A high COT value indicates low physiological efficiency.

#### 2.3.4 Orbital stability

To evaluate orbital stability, we computed FM using a time series analysis that was well established in previous studies [29,56]. We first constructed a time series of 14 dimensional state vector that contains 3 DOF angles of the hip, flexion/extension of the knee and 3 DOF angles of the ankle of both limbs [37,57]. We then defined the beginning and end of one cycle as the heel strike of right leg and the subsequent heel strike of the same leg, respectively; we anchored Poincaré section at the right heel strike. This set-up enabled us to construct a Poincaré map whose input and output are respectively 14 dimensional vectors measured at the beginning of one stride and the next stride;

(Eq. 7) $$S_{k+1}=f\left(S_{k}\right)\text{,}$$

where *f* and ***S***_*k*_ denote the Poincaré map and the state vector at *k*th cycle, respectively.

In theory, the input or output of the Poincaré map during a periodic motion, which we denote as S*, becomes a fixed point of the map and always satisfies

(Eq. 8) $$S^{*}=f\left(S^{*}\right)\text{.}$$

In actual data with various sources of noise and perturbation, the exact S* is unknown, so the average of the state vector is typically chosen as an approximation of S* [57]. Following this conventional process, we selected S* as the average joint configuration at the heel strike across all strides.

After we determined S*, we constructed a time series of error vectors by subtracting S* from each ***S***_*k*_. Then, we constructed a linear map that best approximated the relation between the errors at the neighboring cycles:

(Eq. 9) $$\left[S_{k+1}-S^{*}\right]=J\left[S_{k}-S^{*}\right]\text{,}$$

where **J** is the Jacobian matrix that can be obtained using the least squared error method. For example, if we collect the data of 300 strides, then we can define 14 dimensional error vectors at each right heel strike, ending up with a total of 300 error vectors with the dimension of 14. Then, with the unknown constant matrix **J**, we can formulate 299 vector equations which relate the error vector at *k*th stride with the error vector at (*k*+1)^th^ stride (Eq. (9)). Therefore, the number of unknowns becomes 14 × 14, or 196, whereas the number of equations becomes 14 × 299, or 4186. Typically, there is no **J** that satisfies all the equations, but we can always find **J** that best approximates all the equations or minimizes the squared error.

The FMs are the eigenvalues of **J**, which are typically complex numbers, and their magnitudes indicate the rate of the divergence or convergence under small perturbation [58]. The eigenvalue with the largest magnitude, or max FM is particularly important. For a system to be stable, the max FM should stay inside the unit circle in the complex plane. If max FM is inside the unit circle and the system is stable, then the smaller value of max FM indicates the faster convergence or stronger orbital stability. Following the described procedure, we estimated max FM of each runner using the joint angles at the moment of heel strike.

### 2.4 Statistics

Although insufficient number of cycles can cause biased estimation of FM, the bias becomes negligible when the length of time series is greater than 200 [27]. The pilot test confirmed that 20 min of running typically contain more than 1700 cycles. To ensure reliable estimation of FM and to analyze changes in stability over time, we divided the 20-min run into 4 sections; P1, P2, P3 and P4 respectively indicate the time interval from 0 to 5 min, from 5 to 10 min, from 10 to 15 min, and from 15 to 20 min. Each section contained data of around 400 strides, enabling unbiased estimation of FM [27]. This approach also allowed us to observe changes in the orbital stability depending on running time. We evaluated the mean and the standard deviation of FM for each 5-min interval. Other variables, such as COT and *E*_*norm*_, were also calculated for each 5-min interval to provide a comprehensive analysis of the dependence of both stability and efficiency on running time.

We performed a two-way mixed model analysis of variance (ANOVA) with two groups (Professional vs. Novice) and four time intervals (P1, P2, P3 and P4). We selected mixed-model ANOVA for statistical analysis to investigate both between-subject factors (groups) and within-subject factors (time intervals). By applying the mixed-model ANOVA, we also aimed to handle the inherent correlation in repeated measures, ensuring accurate and reliable statistical results [59]. We used Bonferroni test for post-hoc pairwise comparisons. We used Mauchly's test to check whether the assumption of sphericity was valid. We used Greenhouse-Geisser correction to decrease DOF when the assumption of sphericity was violated. To perform all these statistical analyses, we used SPSS software (IBM Inc., Chicago, IL, USA). The statistical significance level was set at p < 0.05.

## 3 Results

**Fig. 3:**
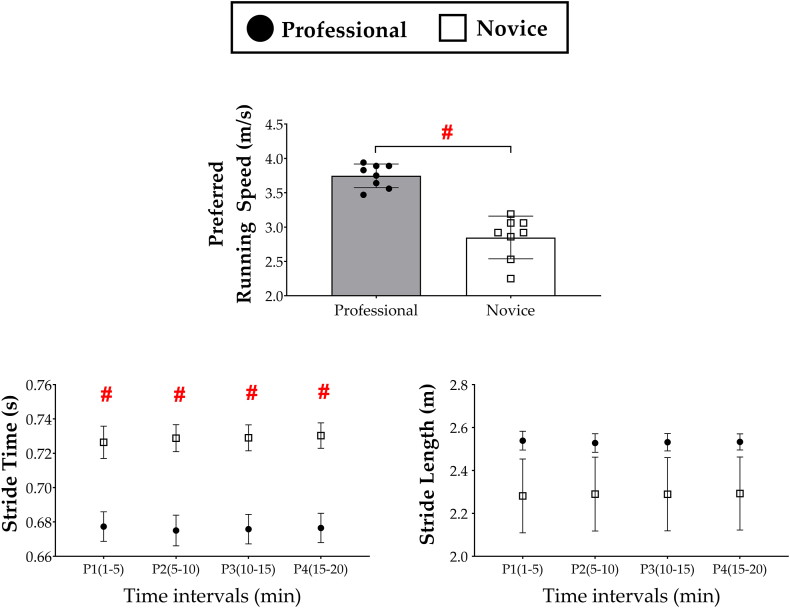
**The preferred running speed and spatiotemporal variables evaluated for the professional runners and novices.** The black circle (●) and white square (□) indicate the mean values of the professional runners and novices, respectively, and the error bar indicates standard error. The hash (#) indicates statistically significant difference between the professional runners and novices (p < 0.05).

All participants completed the running task at PRS. The mean and the standard deviation of PRS of the professional runners were 3.75 m/s and 0.17 m/s, respectively, whereas those of the novices were 2.85 m/s and 0.31 m/s (Fig. 3). The independent samples *t*-test concluded significant difference in PRS between the professional runners and novices [*t*_(14)_ = 7.14, *p* = 0.001]. The effect of group and time on other spatiotemporal variables are also summarized in Fig. 3. The mixed model ANOVA discovered main effects of group on stride time [*F*_(1, 14)_ = 20.23, *p* = 0.001, ηp ^2^ = 0.59]. On the other hand, there was no group difference in stride length. The mixed model ANOVA concluded neither a significant effect of time on the spatiotemporal variables nor an interaction between the group and time for those variables.

**Fig. 4:**
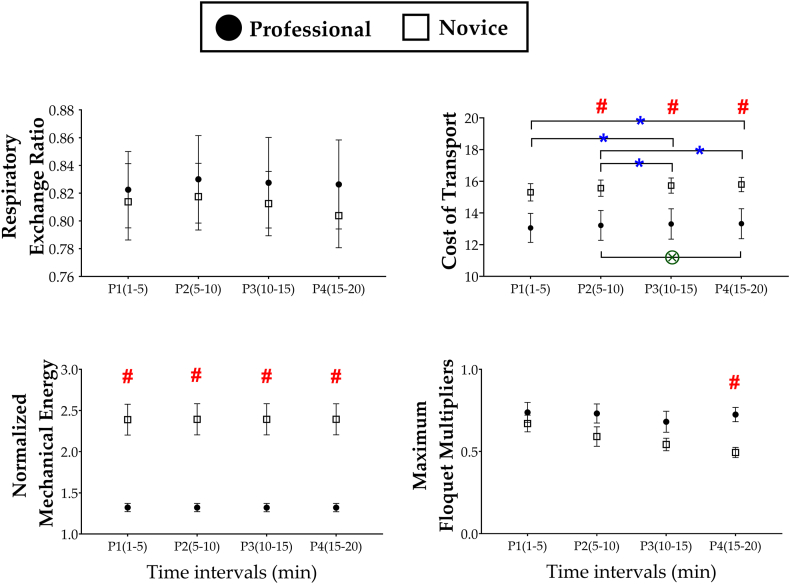
**The exercise intensity, physiological efficiency, mechanical efficiency, and orbital stability evaluated for the professional runners and novices.** The black circle (●) and white square (□) indicate the mean values of the professional runners and novices, respectively, and the error bar indicates standard error. The symbols of * and Ⓧ indicate statistically significant difference between the time interval for the novices and professional runners, respectively. The hash (#) indicates statistically significant difference between the professional runners and novices (p < 0.05).

Fig. 4 shows the mean and the standard error of RER, COT, *E*_*norm*_, and max FM of each group during each time interval. The mixed model ANOVA concluded no group difference in RER, no main effect of time on RER, and no interaction between the group and time for RER; both groups went through similar levels of exercise intensity, and those levels were roughly maintained throughout 20 min. On the other hand, the mixed model ANOVA concluded significant main effects of group [*F*_(1, 14)_ = 4.99, *p* = 0.042, ηp ^2^ = 0.26] and time [F _(1.305, 42)_ = 12.31, *p* = 0.001, ηp ^2^ = 0.47] on COT. The professional runners ran with significantly lower COT or higher physiological efficiency. The independent samples *t*-test concluded that COT of professional runners was significantly lower than that of novices during P2, P3, and P4. No significant interaction between time and group was observed for COT. However, pairwise comparisons concluded significant differences in COT only between P2 and P4 for the professional runners whereas significant differences in COT were observed between P1 and P3, between P1 and P4, between P2 and P3, and between P2 and P4 for the novices.

The mixed model ANOVA concluded a significant main effect of group on *E*_*norm*_ [*F*_(1, 14)_ = 30.11, *p* = 0.001, ηp ^2^ = 0.68], indicating that professional runners ran with significantly higher mechanical efficiency. The ANOVA concluded no main effect of time and no interaction between the group and time for *E*_*norm*_, and the independent samples *t*-test discovered significant differences across groups; *E*_*norm*_ of professional runners was significantly lower than that of novices during every time interval.

For the orbital stability, which was quantified using max FM, the mixed model ANOVA concluded a significant main effect of group [*F*_(1, 14)_ = 8.09, *p* = 0.013, ηp ^2^ = 0.37], indicating professional runners ran with significantly higher value of max FM compared with the novices. The ANOVA concluded no significant main effect of time and no interaction between group and time for max FM, and the independent samples *t*-test revealed that max FM of professional runners was significantly higher than that of novices during P4.

## 4 Discussion

Both efficiency and stability are essential for successful performance in long distance running [19,20]. However, whilst numerous studies have reported the superior running efficiency of trained runners [[2], [3], [4], [5],^7^,^8^,^10^,^12^,^34^,^55^,[60], [61], [62]], no study has assessed their orbital stability during running. In this study, we investigated the orbital stability of running of professional long-distance runners for the first time, adopting the well-established method used in nonlinear dynamics and biomechanics. We observed that max FMs of professional runners are larger than those of novices, which suggests that professional runners exhibit weaker orbital stability of running compared to novices. This result suggests that even professional runners, despite their experience and training, need to sacrifice orbital stability to maintain high running efficiency rather than managing to achieve both high efficiency and strong stability. However, the results support our hypothesis partially; although professional runners consistently exhibited higher efficiency and lower stability (Fig. 4), the statistically significant difference in max FM between the professional runners and novices was observed only for the last 5 min of the 20 min-run.

Professional runners' inability to maintain both efficiency and stability superior to novices seems to contrast with their other capabilities acquired from long training. Previous studies have reported professional runners' ability to adjust running kinematics [[1], [2], [3]], maintain homeostasis, minimize variability [4,5], and maintain a constant COT for a wide range of running speed [[10], [11], [12], [13]]. Therefore, the weaker orbital stability of running of professional runners is plausibly the inevitable consequence of their highly efficient running. Physically, the increase in stability cannot be accompanied by the increase in efficiency because convergence to the planned kinematics under perturbation essentially requires energy dissipation [17]. The observed trade-off between efficiency and stability in professional runners may reflect a physically unavoidable trade-off between the two.

If we regard this trade-off between the efficiency and stability as a constraint, the relatively weak orbital stability of professional runners can be interpreted as another achievement through training and experience. The faster convergence to a limit cycle, the stronger stability, or the lower max FM values all imply aggressive rejection of any unactuated dynamics including the beneficial effect of inertia. If a locomotor system is unable to exploit the beneficial effect of inertia, the system had better conservatively choose inefficient but stable locomotion. The inefficient running of novices can be viewed as such case. Conversely, as in the case of professional runners, if a system is capable of taking advantage of beneficial effect of inertia (possibly through the long training), the system can achieve higher efficiency. Ihlen et al. actually reported that the stability of walking of fallers is stronger than that of non-fallers [63]. Considering the important trade-off between the energy efficiency of locomotion and the strength of the stability under small enough perturbations, it is highly plausible that non-fallers are able to maneuver and take advantage of less stable but more energy efficient walking, whereas fallers assign priority to stronger stability with the cost of less efficiency. Obviously, those who have a high risk of fall walk more discretely than the young and healthy; they choose more stable but inefficient way of walking rather than exploiting the inertia from the previous stride. From this perspective, the more efficient but less stable running of professionals can be regarded as the evidence of higher capability of exploiting useful inertia.

The fact that statistically significant difference in orbital stability between professional and novice runners became apparent only after 15 min of running suggests the plausible change in running dynamics caused by the accumulated time of running. Possible factors include the amount of energy expenditure and fatigue. Prolonged running increases energy consumption and induces fatigue, which potentially increases the risk of injury [64]. Fatigue alters running kinematics, affecting body mechanics and stability, particularly in novices [65,66], and this observation is consistent with that novice runners struggle more with inefficient energy management and insufficient ability to adapt to the impact forces during running [67,68]. In contrast, professional runners, with enhanced muscle power and running economy, might mitigate the negative effects of fatigue on stability better than novices; they prioritize energy conservation and exploit mechanisms to cope with fatigue [69,70]. Therefore, the difference between the professional and novices runners can plausibly become more prominent when the accumulated time of running increases.

In this study, we confirmed the efficiency of the running of professional runners in multiple aspects. First, the low COT of professional runners indicates high physiological efficiency. The O_2_ amount taken in during running in a steady state, known as running economy, is commonly used to calculate the efficiency of energy usage throughout the run of a marathon, and the COT is a particularly important physiological index of the running economy [12,71]. Among athletes with similar levels of the maximum rate of O_2_ consumption, training level and age, the difference in COT results in the difference in the performance [61,72,73]. Second, the low *E*_*norm*_ of professional runners indicates high mechanical efficiency. The mechanical energy during running depends on the joint kinematics [25]. Unnecessary moments and forces owing to the excessive shoulder, hip, and arm counter-rotation can impair running ability, and even the lower arm movement needs to be controlled to minimize anterior-posterior and medial-lateral oscillations in the upper body for good running performance [60]. The lower *E*_*norm*_ indicates more efficient employment of DOFs resulting in the decrease in unnecessary energy usage consumed by superfluous limb movements [26]. Therefore, it is commonly considered that the mechanical efficiency also determines the physiological efficiency. A previous study actually attributed inter-individual COT discrepancies to the difference in kinematic parameters and mechanical energy resulting from the motion of the different segments during running [74]. However, another more recent study showed that *E*_*norm*_ depends on the level of training, whereas COT does not [75]; physically, the mechanical efficiency is associated with the physiological efficiency, but one is not a direct measure of the other. Therefore, we need to investigate the efficiency in both aspects, and in this study, the professional runners clearly showed superior efficiency in both.

It is noteworthy that both professional and novice groups kept RER, the indicator of physiological intensity between 0.75 and 0.85 only with a slight variation (Fig. 4). First, these RER values (less than 0.9) indicate that both groups ran with the submaximal metabolic intensity. Second, the absence of significant difference in RER between the professional runners and novices indicates that our result of superior efficiency of professional runners does not result from relatively less intensity of physiological burden on them. Professional runners chose to run with adequate physiological intensity similar to that of novices by choosing substantially higher PRS than novice runners. More specifically, the professional runners performed significantly more strides than novice runners to achieve higher PRS without significant difference in stride length (Fig. 3).

There are several limitations in this study. First, the findings from this study, which was conducted on a treadmill, might differ for overground running since differences exist between the kinematics of overground running and treadmill running [76]. Future studies should consider conducting experiments in overground conditions to validate the findings. Second, this study does not provide high-resolution information on changes in efficiency and stability based on the level of running expertise (professional, semi-professional, novice, recreational, or on years of experience). Future research could focus on these various levels of runners to provide a more detailed understanding. Lastly, the trials in this study were relatively short. Longer trials with half-marathon or marathon durations could more accurately identify the effects of multiple factors on stability and efficiency during long-distance running. However, maintaining a stable skin interface with the marker for an optical motion capture system for hours can be challenging. Future studies could consider using wearable sensors with a stable skin interface secured by straps, as has been done in past running studies [19].

## 5 Conclusion

We found that professional runners, compared with novices, run with higher efficiency but exhibit weaker orbital stability. Our results suggest that professional runners not only achieve physiological efficiency with low COT but also avoid excessive motions of limbs except for the horizontal translation of COM. On the other hand, professional runners tend to show weaker orbital stability or larger values of max FM, which is an index that provides information on how well the inertia is exploited from one stride to the next. Given the tradeoff between stability and efficiency, it is suggested that the highly efficient mechanism of running of professionals can be partly responsible for the observed weaker orbital stability.

## Ethics and consent statement

On October 23 in 2020, the Seoul National University institutional review board approved the study protocol (institutional review board number 2010/003–013), which conformed to the ethics described in the Declaration of Helsinki. We obtained written informed consent from every participant before the beginning of the experiment.

## Funding statement

This work was supported in part by 10.13039/501100010881Industrial Technology Innovation Program (No. 20007058, Development of safe and comfortable human augmentation hybrid robot suit) and Industrial Strategic Technology (No. 20018157) funded by the Ministry of Trade, Industry & Energy (MOTIE, Korea); the grant of the Korea Health Technology R&D Project through the 10.13039/501100003710Korea Health Industry Development Institute (KHIDI) funded by the Ministry of Health & Welfare, Republic of Korea (grant number: HK23C0071); New Faculty Research Fund of 10.13039/501100002380Hanyang University (No. HY-202300000001156); the grants supported by 10.13039/501100003561Ministry of Culture, Sports and Tourism and Korea Sports Promotion Foundation (RS-2024-00396700, and 2023 SportTech Project); and the 10.13039/501100003725National Research Foundation of Korea (NRF) grants funded by the Korean Government (10.13039/501100014188MSIT) (No. RS-2023-00208052).

## Data availability statement

All data generated and analyzed in the current study can be available from Prof. Jooeun Ahn on a reasonable request.

## CRediT authorship contribution statement

**Siddhartha Bikram Panday:** Writing – original draft, Visualization, Software, Methodology, Investigation, Funding acquisition, Formal analysis, Data curation, Conceptualization. **Prabhat Pathak:** Writing – original draft, Methodology, Investigation, Data curation. **Jooeun Ahn:** Writing – review & editing, Writing – original draft, Validation, Supervision, Project administration, Funding acquisition.

## Declaration of competing interest

The authors declare that they have no known competing financial interests or personal relationships that could have appeared to influence the work reported in this paper.
